# Zebrafish as a model for cardiac disease; Cryo-EM structure of native cardiac thin filaments from *Danio Rerio*

**DOI:** 10.1007/s10974-023-09653-5

**Published:** 2023-07-22

**Authors:** Marston Bradshaw, John M. Squire, Edward Morris, Georgia Atkinson, Rebecca Richardson, Jon Lees, Massimo Caputo, Giulia M. Bigotti, Danielle M. Paul

**Affiliations:** 1 Physiology, Pharmacology and Neuroscience, University of Bristol, Bristol, UK; 2 Translational Health Sciences, University of Bristol, Bristol, UK; 3 University of Glasgow, Glasgow, UK; 4 Institute of Cancer Research, London, UK

**Keywords:** Thin filament, Actin, Troponin, Tropomyosin, Zebrafish, Cryo-EM

## Abstract

Actin, tropomyosin and troponin, the proteins that comprise the contractile apparatus of the cardiac thin filament, are highly conserved across species. We have used cryo-EM to study the three-dimensional structure of the zebrafish cardiac thin and actin filaments. With 70% of human genes having an obvious zebrafish orthologue, and conservation of 85% of disease-causing genes, zebrafish are a good animal model for the study of human disease. Our structure of the zebrafish thin filament reveals the molecular interactions between the constituent proteins, showing that the fundamental organisation of the complex is the same as that reported in the human reconstituted thin filament. A reconstruction of zebrafish cardiac F-actin demonstrates no deviations from human cardiac actin over an extended length of 14 actin subunits. Modelling zebrafish homology models into our maps enabled us to compare, in detail, the similarity with human models. The structural similarities of troponin-T in particular, a region known to contain a hypertrophic cardiomyopathy ‘hotspot’, confirm the suitability of zebrafish to study these disease-causing mutations.

**Supplementary Information:**

The online version contains supplementary material available at 10.1007/s10974-023-09653-5.

## Introduction

The thin filament plays a crucial role in the “cross-bridge cycle” and force production in striated muscle. The main components of the thin filament are actin, tropomyosin, and troponin. Muscle contraction is “switched on’’ by a depolarisation of the sarcolemma that releases calcium into the sarcoplasm, Ca^2+^ is then free to bind to troponin. Ca^2+^ binding causes conformational changes modulated by phosphorylation (Marston and Zamora 2020) that underpin the molecular mechanism of regulation. This allosteric binding produces an azimuthal movement of tropomyosin across the face of the actin subunit, revealing weak myosin binding sites. The steric blocking model that characterises this movement was described by John Squire and David Parry shortly after the 1972 cold spring harbour symposium of quantitative biology where data supporting the model was presented (Parry and Squire 1973; Huxley 1972; Haselgrove 1972); its initial discovery and how this model has developed is reviewed in this issue. However, recent cryo-EM structures and molecular dynamics experiments of the thin filament are providing intriguing insight into some of the conformational changes that occur to the regulatory proteins upon Ca^2+^ activation.

### Cryo electron microscopy of the thin filament

Electron microscopy (EM) has played a fundamental role in revealing the structure and function of the thin filament and its regulatory proteins. Rotary shadowed EM images of isolated troponin indicated the ternary complex had a compact head and an extended tail (Flicker et al. 1982). This work and earlier immuno EM work (Ohtsuki 1975) suggested that Troponin T (TnT) formed the extended tail and that the compact head was what is now commonly referred to as the troponin core domain consisting of Troponin I (TnI), Troponin C (TnC) and the rest of TnT. Visualising these components of small molecular weight (~ 70 kDa) on long flexible filaments was a difficult task, but with the application of single particle analysis techniques to EM images of thin filaments in negative stain, the troponin core domain became visible for the first time (Pirani et al. 2006; Paul et al. 2010). These studies demonstrated that single particle analysis performed without the traditional approach of imposing actin’s helical symmetry in the 3D reconstruction process makes it possible to delineate the troponin complex and its mode of binding to actin and tropomyosin albeit at the relatively low resolution characteristic of negative stain. Subsequent, more detailed analysis of EM images of negatively stained reconstituted thin filaments in both active and relaxed states revealed an L-shaped density for the troponin complex (Paul et al. 2017). The troponin density was interpreted in terms of partial crystal structures of troponin (Vinogradova et al. 2005) and a model of the complete troponin complex (Manning et al. 2011).

The recent application of a similar single particle analysis approach to cryo-EM images of the reconstituted human thin filaments has provided significantly higher resolution structures than those obtainable by negative staining, leading to a substantial step forward in our knowledge of this system (Yamada et al. 2020). The maps recapitulate the L-shaped troponin density but also reveal a novel extended conformation where TnT was observed to cross the filament. TnT emerges from the core of troponin, on one strand of actin, and stretches across to the opposite actin strand where it binds to tropomyosin, in contrast to the previous interpretation in which the troponin ‘tail’ originated from the troponin on the same actin strand (Paul et al. 2017). The density which crosses between the actin strands is continuous and visible at a low contour, indicating the flexible nature and lower resolution of this region of the map. This observation has opened discussion on the role that troponin plays; potentially facilitating crosstalk between strands and how that could affect cooperativity in regulation (Tobacman 2021). It is also apparent that TnT not only provides an important molecular tether to tropomyosin but likely contributes to the inhibition of the crossbridge cycle by physically/sterically blocking myosin binding sites.

Docking crystal structures into electron density maps at high and low Ca^2+^ allowed the analysis of the end points, or extremes, of the physiological process of contraction in the human reconstituted system (Yamada et al. 2020). Extended density running up towards the pointed end of the actin filament in the low Ca^2+^ state was attributed to the C terminal of TnI (CTnI). It is suggested that CTnI is in a ‘locked’ position in the low Ca^2+^ state, effectively holding tropomyosin in place and contributing to the steric blocking of myosin binding sites. At high Ca^2+^ no significant density was recovered for CTnI, implying that tropomyosin is released by CTnI which stops its inhibitory effect and in this ‘open’ conformation CTnI is not closely associated with the thin filament. The CTnI switch peptide was modelled to move into the core region however the final 41 residues remain unaccounted for in high Ca^2+^ conditions (Yamada et al. 2020).

At almost the same time cryo-EM structures of native murine thin filaments were published (Oda et al. 2020). They resolved the core domain in a very similar position on the actin filament as observed in the human reconstituted structures. However, the screw symmetry averaging used to strengthen the signal to noise of the troponin molecules prohibited other asymmetric regions of the map from being resolved. The Galkin lab (Risi et al. 2021), were able to resolve the asymmetric arrangement of TnT in their cryo-EM maps of native porcine thin filaments with no imposition of symmetry. In this study different Ca^2+^ levels were also used but at physiological concentrations, and pairs of troponins (i.e. core domains on different strands of actin) were seen to exhibit different conformations while the position and angle of the core domains were similar to that observed in the human reconstituted structures. These recent cryo-EM studies have unambiguously shown us the organisation and orientation of the troponin subunits across species, but there remains a considerable number of questions to be answered.

A lack of high-resolution detail and structural information in several key functional regions, specifically in TnT and TnI, persists. The thin filament is a macromolecular complex with a stable actin filament core and more mobile regulatory proteins, and as would be expected the resolution of the actin density was higher than the more flexible domains of troponin in the Yamada cryo-EM reconstructions. The global resolution at low and high Ca^2+^ concentrations was 6.6Å and 4.8Å respectively, however the local resolution of troponin in both maps was no greater than 9Å. Only with higher resolution reconstructions in which side chain densities are visible will we be able to accurately determine the effect of disease-causing mutations. Many cardiomyopathic mutations occur at the interfaces between troponin and actin, and troponin and tropomyosin (Tobacman and Cammarato 2021) with the majority of known pathogenic hypertrophic cardiomyopathy (HCM) mutations occurring in the C-terminal of TnI and N-terminal region of TnT (TnT1). The region where TnT1 interacts with the tropomyosin overlap is aptly named the HCM ‘hotspot’ (Palm et al. 2001). It is essential that these critically important regions are resolved in experimentally derived maps to facilitate accurate modelling. A significant increase (3.8Å) in the resolution of Tm overlap region has recently been made by the Galkin lab (Risi et al. 2023) providing new insights into the molecular interactions in this region.

Higher resolution structures of even the most flexible regions will be possible with large numbers of high quality cryo-EM images of fully decorated thin filaments. However, the troponin complex itself is known to dissociate from the thin filament (Yamada et al. 2020). Proteolytic fragments of cardiac Tn (I & T) in blood play a major role as biomarkers and indicators of myocardial infarction or the product of a short-term mechanism to adjust cardiac function under stress conditions (Sheng & Jin 2016). Whilst useful in a medical setting this dissociation is a disadvantage when determining molecular structure in situ. Yamada addressed this problem by using a recombinant tropomyosin with an insertion mimicking a post translational modification that stabilises tropomyosin binding to actin in vivo. They also added 21 times the expected stoichiometric levels of troponin during reconstitution to ensure a high level of decoration. Crosslinking was used to stabilise troponin on native murine thin filaments; with isolated thin filaments displaying a high level of decoration, visible through high contrast imaging using a Volta Phase Plate (VPP) (Oda et al. 2020).Whereas a good level of decoration of troponin was indicated without crosslinking in native porcine thin filaments (Risi et al. 2021).

Cryo-EM is able to capture a single or a series of static conformations of a complex and using robust image processing techniques (Scheres 2012, Grant et al. 2018) can uncover structural heterogeneity. However, it is important to consider the intrinsically dynamic nature of the thin filament. Accurate molecular models of structurally stable regions derived from high resolution electron density maps and µs molecular dynamics simulations can be used in concert. A model of the cardiac specific unstructured N-terminus region of TnI was generated, using the human reconstituted data as a starting point, to study the effects of phosphorylation. The authors concluded that upon phosphorylation of the N-terminus of TnI, Tropomyosin becomes biased to steric blocking positions (Pavadai et al. 2022). Recent modelling work like this combined with cryo-EM have advanced our structural biology knowledge of the thin filament and provided a solid basis to interrogate the pathogenic mutations that cause disease.

### Zebrafish as a model for human cardiomyopathy

Over the past decade zebrafish have become a widely used model organism for the study of cardiovascular disease (Asnani and Peterson 2014). Understanding their regenerative properties could provide opportunities for new therapies for cardiac injury and reduction of scar formation post myocardial infarction. However, it is their ready manipulation by gene editing techniques that has made them into a widely used animal model. Compared with rodent models, zebrafish can provide an increased throughput in structure-function studies for known cardiomyopathy genes. Importantly their diploid genome is well conserved among vertebrates, and the availability of both embryonic and adult models makes zebrafish attractive for many areas of study (Dvornikov et al. 2018).

Support for zebrafish as a relevant model for cardiomyopathies has been shown (Shi et al. 2018) which identified that at the gene level, 96% of the affected genes in HCM have a corresponding zebrafish orthologue. The ‘*silent heart’* phenotype (Sehnert et al. 2002) was the first example of a modification in a contractile protein gene. A reduction in TnT expression through modification of TNNT3 led to a non-contracting heart that was lethal by seven days post fertilisation (dpf). More recently the Bakkers lab have designed a heterozygous TNNTA mutation deleting arginine at position 94 and lysine at position 95 of TnT which created progressive cardiac structural changes resulting in heart failure in adults (Kamel et al. 2021). Cardiac remodelling was observed and as early as 5 dpf increased Ca^2+^ sensitivity was detected in embryos. The adult TnT-RK94del zebrafish developed cardiomyopathy like that observed in patients (Kamel et al. 2021).

Determining the effect of such mutations on the molecular structures of the thin filament proteins will provide functional information and unlock specific disease mechanisms. Furthermore, the 3D structure allows us to identify regions that are equivalent between zebrafish and human and those that differ; to aid the design of disease models. The aim of our study was to obtain high resolution cryo-EM structures of zebrafish thin filaments and validate their use as a structural model for human disease. To date only negatively stained isolated zebrafish thick filaments have been studied using three-dimensional electron microscopy which revealed the zebrafish structure to be similar to the mammalian one (Gonzalez-Sola et al. 2014).

## Methods

### Thin filament isolation

14 6-month-old wildtype TL/EK zebrafish (*Danio rerio*) hearts were excised with the ventricle and atrium chambers stored at 4 ^o^C and the bulbus arteriosus removed. Thin filament isolation procedures were based on those used on goldfish (Kensler and Stewart 1989). Tissue was placed into a 5 ml ‘mincing’ solution (Table S1) to wash before being transferred to a fresh 5 ml solution and left stirring overnight. The following morning hearts were transferred into a 5 ml relaxing solution (Table S2) containing mincing solution and 5 mM ATP and 10 mM creatine phosphate to wash, before being transferred to 5 ml fresh relaxing solution and left to stir for an hour. The hearts were then transferred to a 1 ml relaxing solution and homogenised by hand for 1 min using a polypropylene micro pestle in a 1.5 ml microcentrifuge tube. The cell homogenate was then placed into a thermoshaker at 850 rpm for 45 min. The homogenate was then placed in a benchtop centrifuge for 45 min at 18,000 g and the supernatant transferred to a fresh microtube and placed into an ultracentrifuge for 2.5 h at 100,000 g. The supernatant was discarded, and the pellet was gently resuspended into 150 µl of relaxing solution.

### Cryo-EM

Thin filament samples were screened for good levels of decoration; the criteria for good levels of decoration are visible troponin cores at ~ 385Å, this is not expected along the entire length due to the slow helical path that troponin lies on (Paul et al. 2004). Screening is carried out on a Tecnai T12 microscope (Wolfson BioImaging Facility, Bristol) using 300-mesh copper pioloform and carbon coated grids and a 1% uranyl acetate negative staining procedure. Cryo-EM grid preparation was performed using the Leica EM GP2. 4 µl of sample was placed on a 2/1 quantifoil grid, blotted for 1.6s, plunge frozen and stored in liquid nitrogen. A ThermoFisher Talos Artica (200 kV) (GW4 Cryo EM Facility, Bristol) was used to screen the frozen hydrated samples and full acquisition was performed on the ThermoFisher Titan Krios (300 kV) (eBIC, Diamond) using a VPP and EPU automated collection software. Two separate collections were performed using the same microscope both with a pixel size of 1.048Å/p, acquiring 3,105 (TF1) and 2,517 (TF2) micrographs (total 5,622) (Table S3).

### Image processing

The micrographs were collected and imported into Relion 3.0 (Zivanov et al. 2018). Beam induced motion correction was performed using Motioncorr2 (Zheng et al. 2017). Defocus and phase shift parameters were then determined using CTFFIND4 (Rohou and Grigorieff 2015).

For cardiac thin filament reconstruction, the micrographs were manually picked in Relion, with regions of extra density along the actin filament that corresponded to the expected troponin repeat of 385Å selected. 9,664 (TF1) and 8,928 (TF2) particles (total 18,592) were extracted using a box size of 400 pixels and classified using Relion 2D classification. 2D class averages with any apparent troponin and/or tropomyosin density were selected. 9,821 particles were combined and exported into cisTEM (computational imaging system for transmission electron microscopy) (Grant et al. 2018). A 3D reconstruction of the thin filament was calculated from these particles without use of a reference model using a down-sampled pixel size of 5.3Å.

For the cardiac actin reconstruction, 2,606 start-end helix coordinates were manually selected in Relion, allowing extraction of 23,417 particles into 500-pixel boxes, segments were extracted every two asymmetrical units, each of which were 27.6 Å. Helical 2D classification was then used to generate 50 class averages, using a 460 Å mask diameter, 15 of these classes which showed clean actin across the diameter of the box and had clear actin polarity were selected. These class averages served as references in Relion Autopick, which was performed using a 27.6 Å helical rise of 2 asymmetrical repeating units, and a minimum inter-particle distance of 100 Å. Autopicked particles were extracted into 500-pixel boxes, and a secondary 2D classification generated 200 class averages using a 220 Å mask. The final 51 classes, exhibiting clean density and apparent actin polarity, yielded 420,108 particles within 500-pixel boxes. These particles were imported into cisTEM and non-helical Auto Refine was run using a mask radius of 230 Å, a global mask radius of 250 Å, auto masking was also applied, 7 rounds of refinement were completed and the resultant map and final reconstruction half-maps were imported back into Relion. Mask Creation was used to generate a binary helix mask and used for the Post Processing step, which sharpened the map and provided an fsc curve showing an estimated resolution value of 3.85 Å (Fig. S10).

### Obtaining orthologs to human thin filament sequence

Protein sequences were obtained from UniProt. For TnT, TNNT2_HUMAN we identified A8E586_DANRE as the best ortholog in zebrafish (66% sequence identity). For TNC (TNNC1_HUMAN) there were 2 possibilities to choose from and we chose Q800V7_DANRE due to its slightly higher sequence identity (90% identity) than the alternative Q6IQ64_DANRE (88% identity). For tropomyosin we used TPM1_DANRE (92% identity) and for actin F1RCB6 (98% identity). For TNNI3 the choice was more difficult due to various gene duplications between humans and zebrafish and there is no simple orthology relationship. We chose Q5PR62_DANRE which we have used for EM models. However, a more recent version of the sequence is now available (F8W4T8_DANRE) with identical sequence length and 3 AA’s difference at the N-terminus and we suggest future researchers switch to this sequence.

### Modelling

The human cardiac thin filament model 6KN8, (Yamada et al. 2020), was used as a homology model, the software SWISS-MODEL (Waterhouse et al. 2018) was used to replace existing residues and generate a model (QSQE − 0.93) with the zebrafish amino acid sequences obtained from Uniprot (https://www.uniprot.org/). The model was then exported and fit into the zebrafish map using Chimera (Pettersen et al. 2004), structured elements were treated independently, moved into the density and new coordinates reinstated into the pdb to generate the zebrafish model. The ChimeraX (Pettersen et al. 2021) tool ‘Surface Zone’ was used to divide the map into groups. The zebrafish model was used to segment the map using a radius to include the protein density creating a faithful representation of the original map.

## Results

### Native cardiac thin filaments from zebrafish

**Fig. 1:**
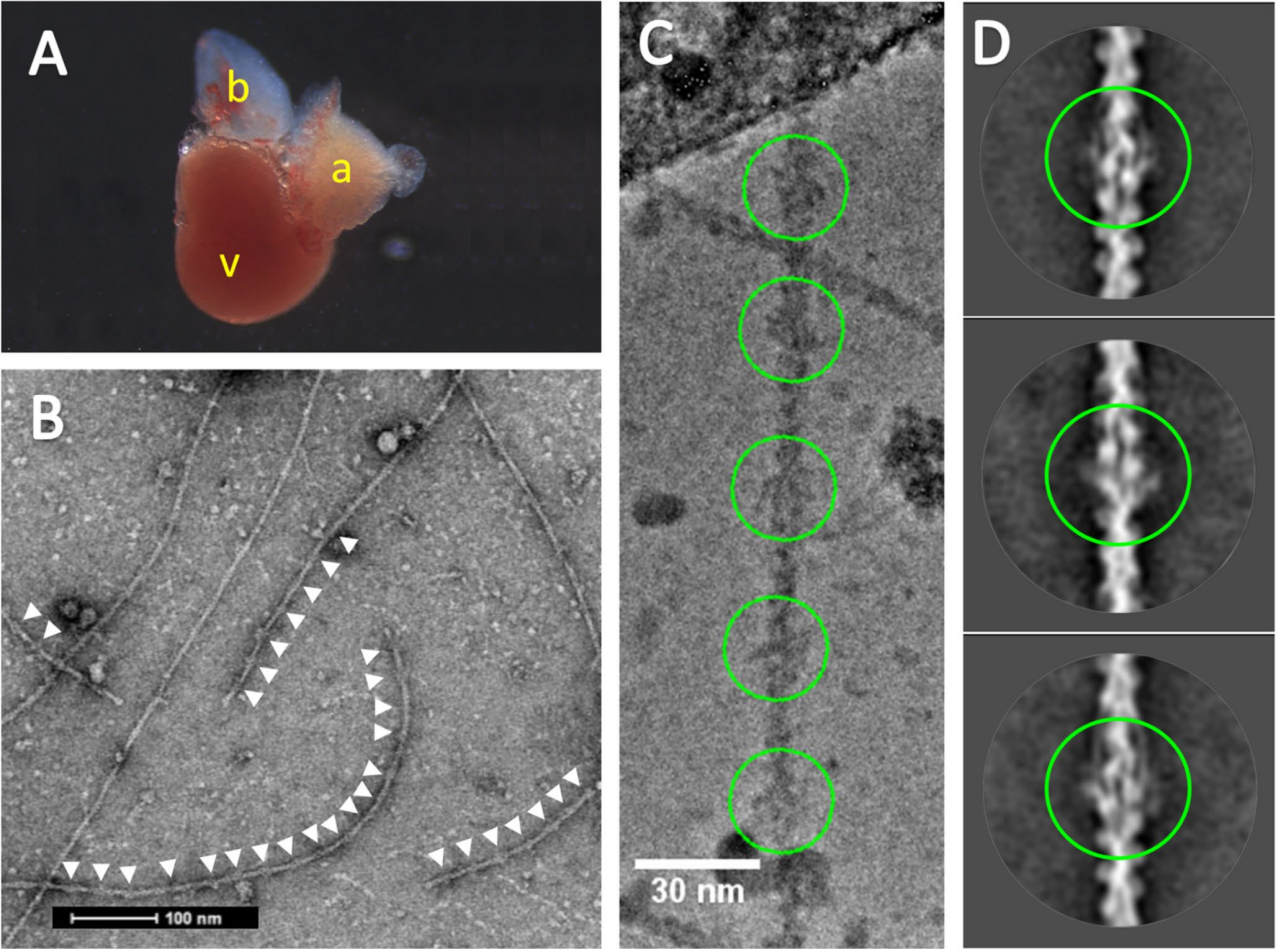
Cryo-EM of zebrafish cardiac thin filaments. **A:** An excised adult Zebrafish heart, letters (in yellow) show V-Ventricle, A-Atrium B-bulbus arteriosus. **B:** A typical negative stain EM micrograph of isolated thin filaments, visible troponin complexes indicated with white arrows. **C:** Cryo-EM image illustrating the periodic binding of troponin on the thin filament (green circles) at a spacing of ~ 385Å **D:** 2D class averages from cryo-EM data with pairs of troponin labelling the two strands of actin (green circles); tropomyosin strands are also visible (top and bottom images)

**Fig. 2:**
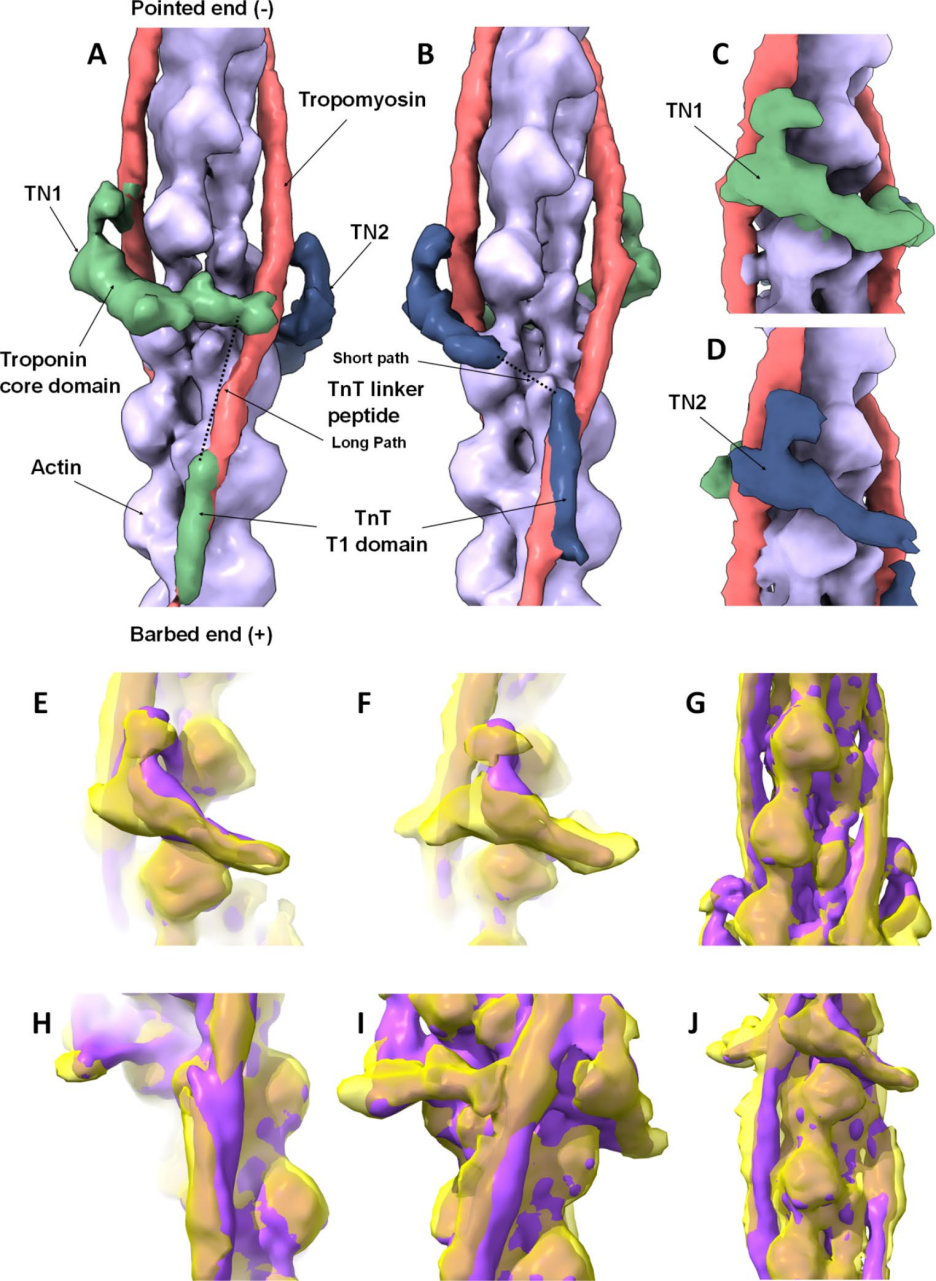
3D reconstruction of native zebrafish cardiac thin filaments and comparison to human reconstituted thin filament structure. **A-D:** Surface rendered protein density map, highlighting the thin filament constituent proteins, segmented and colour coded as follows; Tn1 green, Tn2 blue, tropomyosin pink and actin purple. **A** & **B:** views of the complete map oriented to illustrate the two distinct paths taken by individual troponin molecules on each side of the thin filament as they span the two tropomyosin strands. A & B are related by 180^o^ rotation about the central axis of the thin filament. The different paths of troponin are apparent; Tn1 is located higher on the filament than Tn2, however, the TnT linker peptide path of Tn1 to tropomyosin is longer than that of Tn2. More density is recovered for Tn1 despite its linker peptide following the longer path. **C** & **D:** Close up views of the troponin core domain illustrating its rotated ‘L’ shape which is consistent on both sides. Views as in A & B but rotated by 60^o^ about the central axis. Segmented regions calculated using ChimeraX. The pointed (-) and barbed end (+) of the actin filament are indicated. **E**–**J:** The zebrafish (yellow) and Yamada (purple) high Ca^2+^ state reconstructions are superposed. **E** & **F:** Extra protein density in Tn1 and Tn2 core regions is visible in the zebrafish map. **G** & **J:** Tropomyosin and actin densities are similar. **H:** Strong similarity in TnT T1 domains interacting with tropomyosin overlap region. **I:** Extra TnT linker-region density in the zebrafish map not present in other thin filament maps

**Fig. 3:**
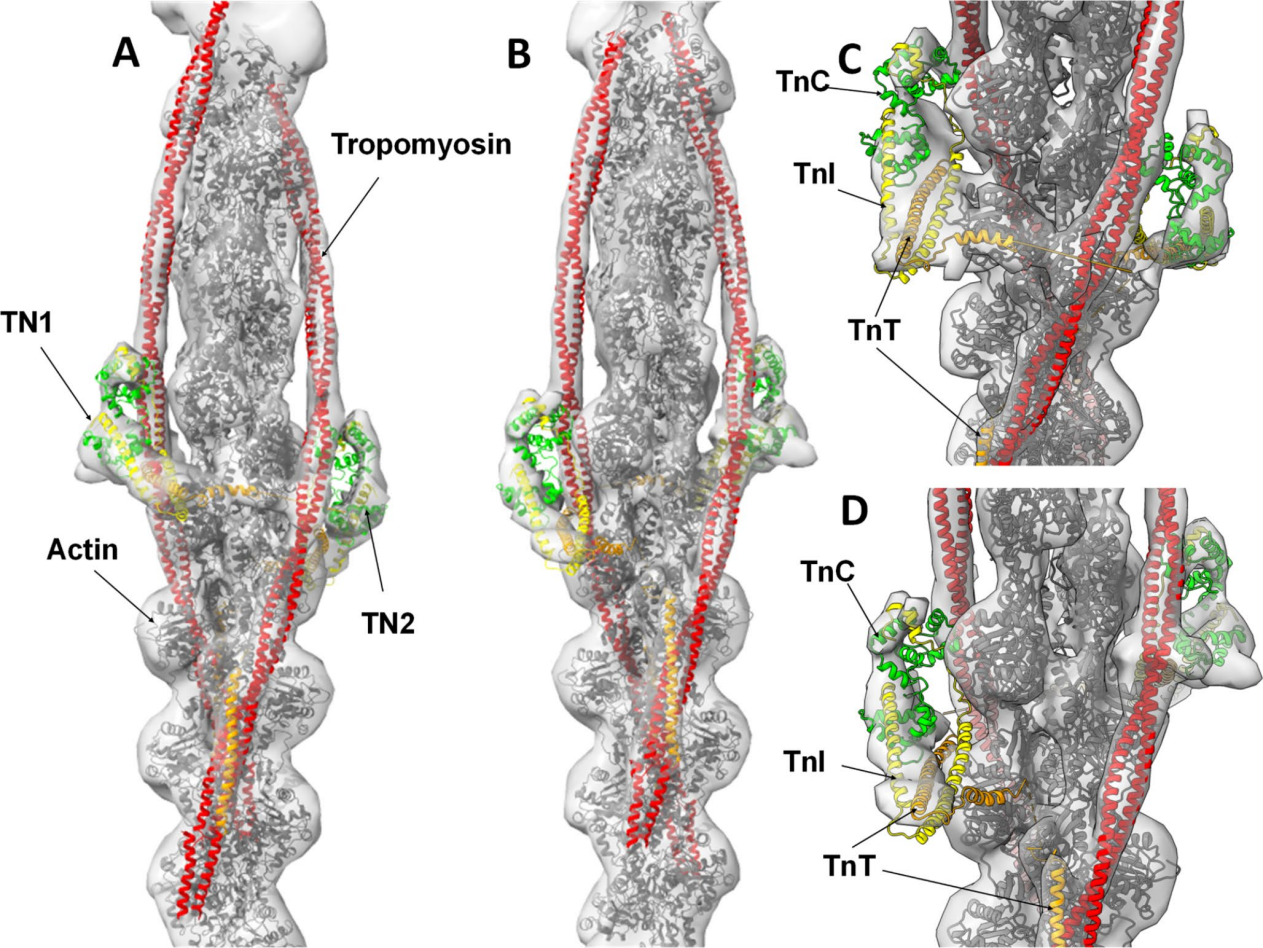
Zebrafish high Ca^2+^ thin filament model. The full-length actin (grey), TnC (residues 2-161, zebrafish 2-161) green, TnI (41–166, zebrafish 10–135) yellow, TnT (99–272, zebrafish 101–207) orange, full length tropomyosin red, atomic models docked into the electron density. **A** & **B:** 180° rotations, **C** & **D:** close up of the central region with 180° rotations

**Fig. 4:**
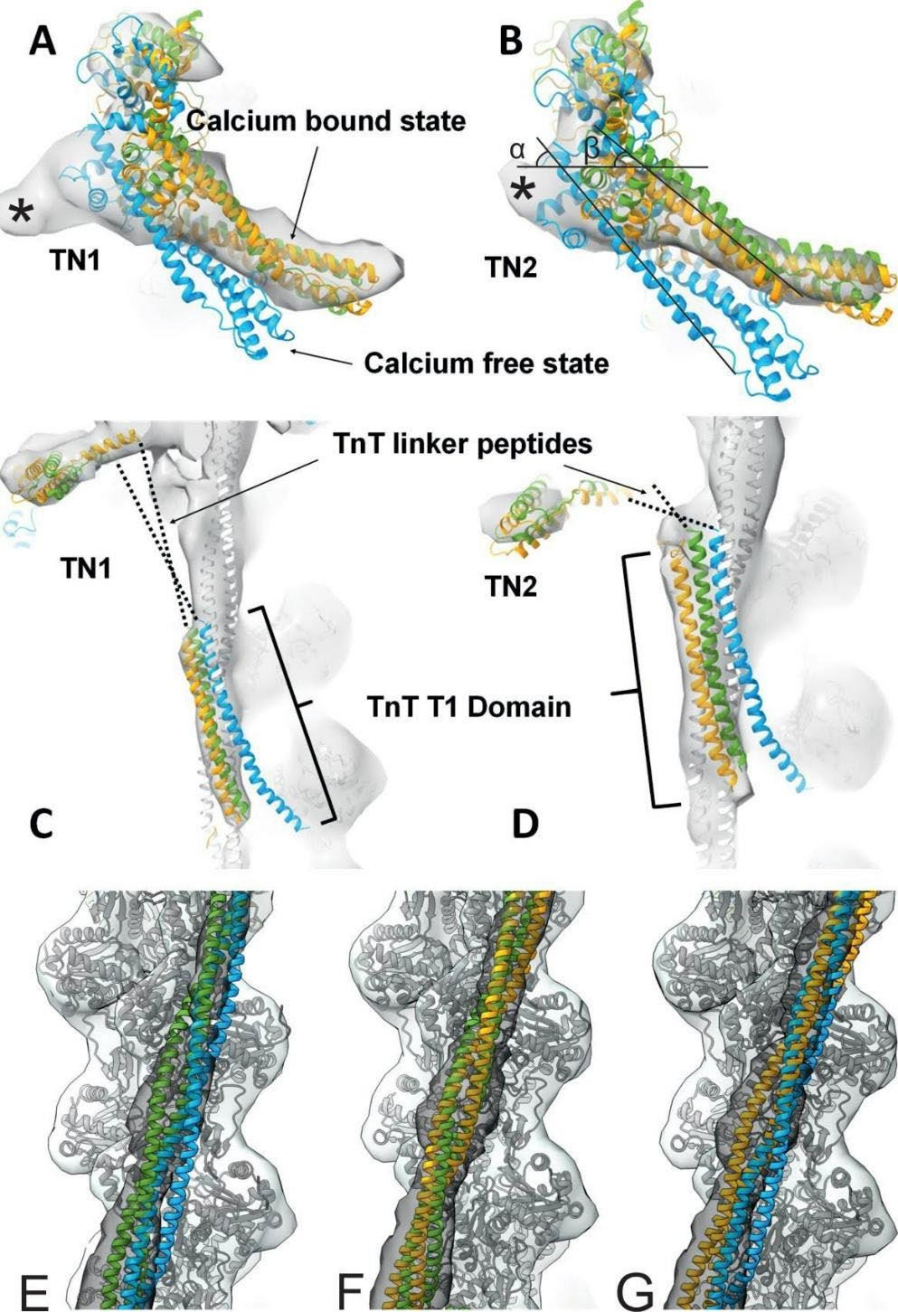
Conformation of troponin core domain and tropomyosin positions. The high and low Ca^2+^ models 6KN8 (green) and 6KN7 (blue) were docked into our thin filament reconstruction and compared to our zebrafish model (yellow). **A** & **B:** the core domains of Tn1 and Tn2. Regions of empty density are indicated with *. **B:** ɑ (45°) & (35°) the angle the TnI helix 1 makes with the horizontal in the two states **C** & **D:** the tropomyosin overlap region where TnT1 domain is located of Tn1 and Tn2 respectively. **E,F,** & **G:** Pairwise comparison of the position of tropomyosin from the three models

The zebrafish thin filaments in this study were isolated directly from the adult cardiac muscle, thus the native filaments were subject to any naturally occurring post translational modifications. Additionally, no cross-linking or modifications were used to enhance the stability of the regulatory proteins or flexible domains. Cardiac muscle from 14, 6-month-old wild type zebrafish (Fig. 1A) were pooled to obtain sufficient tissue for the isolation. The use of the VPP (Fig. 1B) allowed for the identification of filaments decorated with troponin and tropomyosin (Fig. 1C). However, the percentage of decorated filaments actin was ~ 10%, as discussed above: the dissociation of the thin filaments upon isolation is a known issue (Yamada et al. 2020). Protocols to isolate zebrafish myosin filaments were adapted (Gonzalez-Sola et al. 2014) and manual selection of decorated thin filaments carried out using previously established techniques (Paul et al. 2010; Yang et al. 2014). The level of decoration of negatively stained thin filaments can be seen in Fig. 1. Particles were centred on the troponin complex and with only ~ 10,000 particles we reconstructed a 480Å long segment of the thin filament (EMD-15,901). The Fourier shell correlation was calculated from two independent half maps to obtain an estimated overall resolution of 15.4Å for the final map (Fig. S1). As expected, the more rigid structure of the actin backbone is better resolved than the flexible linker regions of troponin, this is shown in Fig S2 where the reconstruction has been filtered to the local resolution. To avoid over interpretation of the density attributed to the regulatory proteins in Figs. 2, 3 and 4 the map was filtered to 15Å. A comparison of the human reconstituted reconstruction filtered to 15Å resolution is shown in Fig. S3.

The regulatory proteins troponin and tropomyosin are readily identified in our reconstruction, troponin lies over two adjacent actin subunits on the genetic left-handed actin helix, leading to a 27.5 Å axial rise between the globular core regions and the characteristic troponin stagger (Fig. 2A-B). Crucially, the density linking the globular core to the opposite tropomyosin strand is recognised as the extended tail of troponin based on the assignment of troponin components made in the human reconstituted and native porcine structures (Yamada et al. 2020) (Risi et al. 2021). The stagger of the globular core domain combined with the ability of troponin to bridge the actin and tropomyosin strands results in a fully asymmetric macromolecular complex. Both troponin ternary complexes have the same rotated ‘L’ shape; with the long arm of the ‘L’ nestled between actin subunits. The general ‘L’ shape is similar to that seen in our previous negative stain structures (Paul et al. 2010, 2017). More density is recovered for the upper troponin complex (Tn1) in the zebrafish map, with additional density extending from the long arm of the ‘L’ into the TnT linker peptide region. This additional density enhances the overall asymmetry of our reconstruction in the troponin region. This difference which we observe between Tn1 and Tn2 may arise from stabilisation of Tn1 flexible linker peptide due to local interactions in this position which may not occur in the Tn2 linker peptide in the equivalent region on the other side of the thin filament. Conformational differences between tropomyosin core domains on different actin strands have also been reported and attributed to different Ca^2+^ states, but without differences in this linker region (Risi et al. 2021).

The TnT linker peptide crosses from the core domain on one strand and connects to the T1 domain that runs down the tropomyosin strand, towards the barbed end of the filament. Due to the stagger of the troponin core regions each linker peptide takes a path of different length. Explicitly, the path of Tn1, the upper of the two complexes, crosses to the lower of the two T1 domain-tropomyosin overlap regions, resulting in a longer path than for Tn2. We have recovered density for the T1 domain-tropomyosin overlap regions of both Tn1 and Tn2 (Fig. 2B & D). The variation in the length of the path of TnT seen in this map is a feature of the human reconstituted and native porcine structures (Yamada et al. 2020; Risi et al. 2021) and is the subject of recent study employing time-resolved fluorescence imaging & computational modelling (Deranek et al. 2022).

The zebrafish and both the Yamada high and low Ca^2+^ human thin filament maps are shown filtered to 15Å in Fig. S2. The zebrafish map clearly resembles the high Ca^2+^ human thin filament map more closely than the low Ca^2+^ structure. The angle at which the long arm of the L-shaped core domain emerges from the filament was consistent with the high Ca^2+^ map; this is even more apparent when the two structures are directly superposed (Fig. 2I-J).

A full (length) homology model of the high Ca^2+^ zebrafish thin filament was calculated in SWISS-MODEL (see methods) using zebrafish protein sequences. The final model was composed of actin, tropomyosin, TnC (residues 2-161), TnI (residues 41–166) and TnT (residues 10–135). The fit in map utility in Chimera gave a good initial alignment of the entire complex as a rigid body within the density. An improved manual rigid body fit of structured elements was performed. The manual fit of each troponin complex was independent as the full thin filament is asymmetric and no symmetry operators could be used. The refined fit was good (51,170 of 60,788 atoms inside contour (threshold − 0.875)) with a correlation score of 0.8, for comparison the Yamada high Ca^2+^ model in the zebrafish electron density gave a correlation score of 0.79.

The N-terminal lobe of TnC is an area of weak density in the zebrafish map, with the atomic model lying outside the electron density; despite this the similarity of the zebrafish map to the Yamada map and the overall quality of fit into the density establishes the agreement with the human thin filament structure.

### Tropomyosin and troponin in a high Ca^2+^ state

The azimuthal position of tropomyosin typically indicates the activation state of the thin filament. The binding of Ca^2+^ to TnC is known to precipitate the movement of tropomyosin across the actin subunit revealing first the weak then the strong myosin binding sites. To that end we inspected the azimuthal position of tropomyosin to determine the activation state of our thin filament structure. Whilst it was not possible to resolve the individual strands of tropomyosin, the density was sufficient to track the path of the coiled-coil. The full-length tropomyosin molecule was docked into the density as a rigid body. The position of tropomyosin was found not to block weak myosin binding sites and in good agreement with the 6KN8 Yamada high Ca^2+^ state. Chopping tropomyosin into shorter segments would provide the ability to interrogate the molecule’s flexibility further (Rynkiewicz et al. 2022; Paul et al. 2017) as would higher resolution reconstructions of these flexible regions. The differential movement of tropomyosin that we reported in 2017 could have been due, in part, to low resolution density contributions from TnT, which is now known to cross strands. Through these docking experiments we were able to establish that the thin filament in our map corresponded to a high Ca^2+^ state. This result was unexpected due to the isolation protocol being carried out in a relaxed state, potential reasons for this are discussed below.

Further rounds of 3D classification were carried out on the data to determine whether there was an underlying mixed population of Ca^2+^ states. The data was sorted into two 3D classes and then four; in both cases the resultant 3D reconstructions returned a consistent tropomyosin position and location of the IT arm (Fig S5 & S6). This analysis gave no indication of a population of relaxed filaments within the data. However, minor populations of single sided troponin filaments were observed, this heterogeneity in the data will have reduced the signal to noise and the resolution, of the troponin density.

The fit of the different models into the globular core domain were critically assessed. The IT arm, which corresponds to the long arm of the L shaped motif of the troponin complex, and which is made up of a α-helical coiled-coil structure formed from part of cTnI and cTnT, makes an acute angle with the horizontal in both models, sloping down away from TnC (Fig. 4B). By comparing the angle of helix 1 in TnI in both human models, we observe that this angle is greater in a low Ca^2+^ state (6KN7) ~ 45° than in the high Ca^2+^ state (6KN8) which intersects the horizontal at a shallower angle of ~ 35° (Fig. 4A & B). The same helix in the zebrafish model runs parallel to that in the high Ca^2+^ state model (6KN8). Whilst the angle of the zebrafish IT arm closely aligns to that of the human model at high Ca^2+^, it is known that in cardiac muscle it can be mobile and as such not necessarily an indicator of activation state (Sevrieva et al. 2014). If the Tn-I switch peptide had been resolved to a higher resolution a more definitive determination of the regulatory state could have been made; by considering interaction with TnC in the closed state and with tropomyosin and actin in the blocked state.

Both globular lobes of TnC are more closely associated with the actin filament in the human high Ca^2+^ model (Yamada et al. 2020); this was also true of the zebrafish map density. The N-terminal domain of TnC in the zebrafish map, particularly in TN2 (Fig. 3A &C), is a region of reduced density, and neither of the Yamada models fit well in this region. However, the high Ca^2+^ pdb is contained within the molecular envelope of the zebrafish map to a greater extent. Thus, both the position of tropomyosin and the conformation of troponin in the zebrafish thin filament match the high Ca^2+^ conformation of the human thin filament.

### Similarities and differences between the zebrafish and human thin filament structures

The pairwise alignments of the contractile proteins show a substantial level of sequence identity between the species (Fig S7). This is with the exception of the human N-terminal extension of TnI, which is missing in zebrafish. This region has not been resolved in the human structures and is thought to be flexible. The zebrafish map has a region of unassigned density at the base of the core adjacent to the C-lobe of TnC (Fig. 4A & B indicated with *). The proximity of this density to tropomyosin and its shape resembles the molecular dynamics modelling of the N-terminal extension of cardiac TnI (Pavadai et al. 2022). Whilst density for this region would be expected in human maps, zebrafish have no equivalent region. A BLAST search was carried out of the whole zebrafish genome to establish whether there may be an additional protein that served a similar physiological role, but no candidates were found. In humans the phosphorylation of serine 23 and 24, located in the N-terminal extension, reduces Ca^2+^ sensitivity which corresponds to an increased rate of relaxation and heart rate during normal response to cardiac stressors like exercise. It is conceivable that zebrafish do not need such an adaptation.

Zebrafish Troponin T is 16 amino acids shorter than human, with a small number of deletions including a 6 amino acid truncation of the C-terminus. It is likely that the small 2–4 amino acid deletions dispersed along the protein are uninterpretable in the context of our map. The extra density resolved in the zebrafish map for the linker region of TnT1 that has no equivalent density in the human maps corresponds to 161–208 in the sequence. This region has a 63% sequence identity with human, (compared to 68% for full length TnT) the slightly reduced identity the sequence does not provide us with any indication of the origin of this density. It may be indicative of a stabilising local interaction or as these are native filament preparations potentially the contribution of additional proteins. The skeletal muscle protein nebulin was recently observed to bind to the TnT linker region (Wang et al. 2022).

Other differences in the zebrafish reconstruction compared to the human is in the N-lobe of TnC, with reduced density found in the zebrafish map. There is no elucidation to the reason for this on the sequence level, with the identity between these two proteins being 90.1%, no deletions and the same length in both species. It is likely the reduced density is in part due to a less well resolved mobile region.

The architecture and orientation of the troponin core domains in human (Yamada et al. 2020) and zebrafish are consistent and reversed from the interpretation made in previous studies of negatively stained thin filaments (Paul et al. 2017; Yang et al. 2014). The different interpretation made in these studies most likely reflects the lower resolution characteristic of negative stain as well as the difference between earlier predictions of the overall architecture of the troponin complex and the unexpected arrangement identified in the cryo-EM analysis. All recently published cryo-EM maps of the thin filament have the core domain oriented in the same way demonstrating not only the step change in resolution of the thin filament given by cryo-EM but also the structural similarities across human, porcine and murine orthologues. Of note is that the orientation of the IT arm is very close to that described in an in-situ polarised fluorescence study of skeletal muscle (Knowles et al. 2012); however more flexibility was then described in the IT arm of cardiac muscle by the same lab (Sevrieva et al. 2014).

We report that both ternary troponin complexes in our reconstruction appear to be in the same state, with a very similar arrangement of main helices of TnI, C & T within the core domain. We attribute the slight differences in the N-terminal lobe of TnC to weak signal and low resolution in this area. No evidence of a differential behaviour of troponin within the core domain ‘pair’ was seen here; this was also the case as in the human thin filaments. However, this may not be expected unless prepared in a more physiological range of Ca^2+^ levels (Risi et al. 2021) and not resolved without a significant improvement in the resolution of the troponin complex.

The tropomyosin overlap region and its interaction with TnT is readily identified in our zebrafish map as in a similar manner to the human and porcine structures indicating this region’s importance and functional significance across species. The two distinct paths of TnT crossing the actin strands and giving rise to links between the two tropomyosin strands were also seen here. The mechanistic significance/implications of this asymmetric arrangement are yet to be fully understood. Zebrafish tropomyosin has a very high sequence identity (92%) with human cardiac, due to the limited resolution in our map no differences were resolved.

**Fig. 5:**
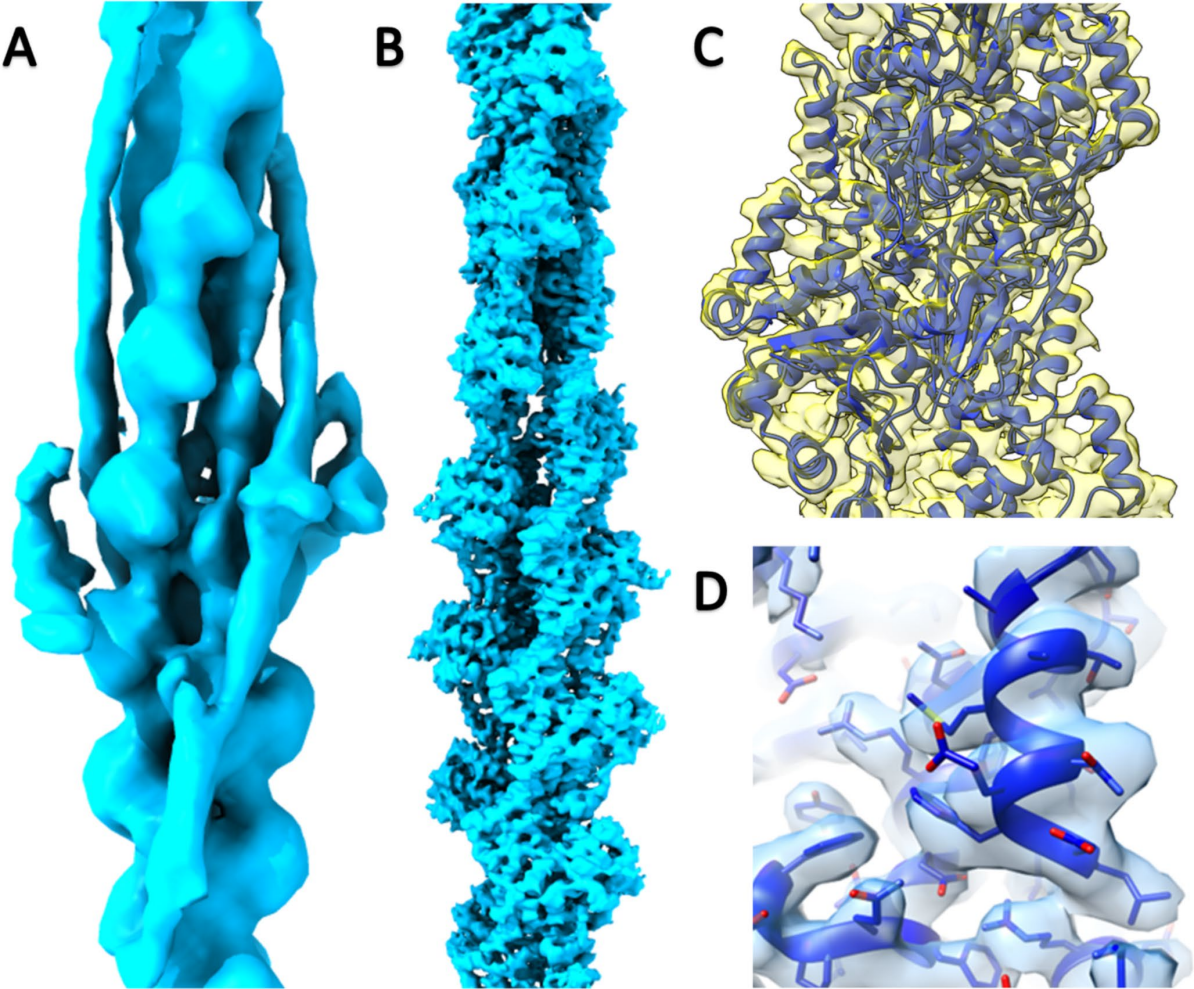
Cryo-EM reconstructions of the zebrafish thin filament and actin filament. **A:** The complete asymmetric unit of the thin filament composed of 14 actin subunits, 2 tropomyosin and troponin complexes. **B:** An equivalent region of the actin filament composed of 14 actin subunits. **C:** Detailed region of the actin filament map (yellow) with cartoon representation of the fitted coordinates (blue). **D:** Detailed region of an individual actin subunit protein density with side chains coordinates represented as sticks

Zebrafish actin has, as expected an extremely high sequence identity (98%) to human. Our data contained large numbers of undecorated actin filaments providing the opportunity to determine the structure of zebrafish cardiac actin. Without the imposition of helical symmetry, we calculated an actin structure of equal length to our thin filament map to a resolution of 3.89Å, (EMDB 17120) (Fig. 5.). This significantly higher resolution provides the ability to assess the equivalence of our docked zebrafish model (PDB 8ORD) to human F-actin structure (6KN8) with more precision. A TM score (Zhang and Skolnick 2004) of 0.97 and a RMSD of 1.19 confirms the expected structural similarity of the two proteins (Fig S11).

Through a detailed comparison of the zebrafish and human reconstituted thin filament structures we can confirm that the contractile apparatus is broadly structurally equivalent. This study has revealed the zebrafish structure in a single conformation corresponding to a high Ca^2+^ state, and consequently we cannot report on the structural changes that underpin regulation. However, the implications of the structure being conserved across species is significant, indicating that functional domains and mechanisms are the same.

## Discussion

In this study we determined the cryo-EM structure of the native thin filament in a high Ca^2+^ state, docked a zebrafish homology model into the density and compared this to the human reconstituted thin filament model. We have explored the suitability of zebrafish as a structural model for studying cardiomyopathies.

Models to study the effect of HCM causing mutations in TnT are invaluable tools for research and in the development of therapeutics. Mutations in the cardiac TnT gene (TNNT2) are the most common HCM-causing mutations after MYH7 and MYBPC3; with increased incidence of arrhythmias that cause sudden death (Watkins et al. 1995; Moolman et al. 1997). There has been substantial progress toward our understanding of how HCM causing mutations in MyBPC may cause hypercontractility that proceeds hypertrophy; with small molecule modulators now being used to treat the condition. A clearer understanding of the pathophysiology caused by mutations in TnT will hopefully lead to similar progress. The development of a zebrafish HCM heart failure model is a great step towards understanding the pathological changes that occur by targeting TnT (Kamel et al. 2021). The genetic tractability of the zebrafish can be further harnessed to model precise disease-causing mutations allowing functional in vivo assessments of development and cardiac function along with multiscale imaging of tissue and proteins.

As described above the structure of the zebrafish thin filament is very closely aligned to the human reconstituted thin filament structure, and as such this provides an opportunity to model and investigate the structural implications of human HCM mutations. The Bakkers lab have recently targeted the HCM hotspot (Palm et al. 2001) near the tropomyosin overlap region (Kamel et al. 2021). One of the resultant zebrafish lines had a TnT-RK94del, the heterozygous mutants reached maturity whereas the homozygous embryos did not survive longer than 2 weeks. The heterozygous adult zebrafish developed progressive cardiac abnormalities and went on to develop heart failure and ultimately cardiac remodelling, both of which are phenotypic traits seen in human patients. A deletion in this region would remove the end of the α-helix, disrupting the TnT-tropomyosin interface. Since classically this helix of TnT has been thought to provide increased structural stability to the tropomyosin overlap, this deletion may provide more flexibility (Kamel et al. 2021). The region of highest sequence conservation between the human and zebrafish is at the site of the highest density of HCM mutations in TnT (Fig S8). Because of the high sequence conservation in the hotspot region, it is simple to find the equivalent zebrafish residue and begin to interpret the effects.

The structural rigidity of the TnT overlap helix is seen as well resolved density in all the cryo-EM thin filament maps to date indicating this helix makes a strong interaction. This consistently well resolved region, along with the fact that large numbers of HCM mutations are located here, is further evidence of its essential biological function. However, we have less structural information about other regions of TnT, the N-terminal, for example, is highly glutamate rich and commonly referred to as the hypervariable region. Polyglutamate tracts are known to switch between helix and random coil states depending on pH (Nakamura and Wada et al. 1981). Intriguingly the C-terminal of TnT in Drosophila is also highly glutamate rich, and it is suggested that it acts as a Ca^2+^ store or reservoir, critical for the fast asynchronous contraction of flight models (Cao et al. 2020). The possibility of a similar mechanism could be considered here where the negatively charged region could store or even attract or direct Ca^2+^ towards TnC. Increased local resolution of TnT and the whole thin filament, will enhance our knowledge dramatically.

### Limitations of our study

Our preliminary findings would be strengthened by a higher resolution reconstruction of the full thin filament. The biggest limitation in our study is the lack of particle numbers in our data, with under 10 thousand particles the achievable resolution is highly limited. This is a direct consequence of the lack of fully decorated thin filaments in the frozen hydrated sample. As discussed, the troponin complex readily dissociates from F-actin, with rapid freezing and interaction with the air-water interface thought to enhance this dissociation. Efforts to reduce this dissociation are paramount, looking towards successful mammalian preps may inform our protocols; a faster approach may also reduce protein dissociation. Employing the new generation of freezing techniques will be explored to enhance the quality of our starting data and increase particle numbers. Increased particle numbers will facilitate an examination of any heterogeneous subpopulations that arise and importantly increase the achievable resolution allowing more accurate docking of molecular models.

The unexpected result that our zebrafish thin filaments were in a high Ca^2+^ state when prepared in a relaxing solution could be due to several factors. There may have been insufficient EGTA to chelate the Ca^2+^, leaving enough endogenous Ca^2+^ to activate the filaments: very small quantities of Ca^2+^ are needed to obtain activation (Risi et al. 2021). Although established concentrations of EGTA were used, overall volumes of relaxing solution may have been too low. It is known that there is an equilibrium between states & multiple studies have reported a proportion of their filaments to exhibit the different state to expected (Pirani et al. 2005). It is also recognised that the activated state is more stable and greater stability would be likely to produce more decorated filaments and consequently our particle selection procedures may favour this activated state. Finally, we cannot discount the possibility that zebrafish may have evolved an adapted mechanism of Ca^2+^ activation and/or regulation; superfast kinetics of excitation and coupling has recently been demonstrated in zebrafish skeletal muscle leading to sustained Ca^2+^ transients (Idoux et al. 2022).

Within the last 2 years our knowledge of the structure of the thin filament has changed dramatically. The revelation that the troponin T crosses tropomyosin strands has changed the way we think of the thin filament structure. The structure of the troponin complex in situ has now been revealed to be more complex, with extensions from both TnT and TnI. The full extent or wingspan of the molecule may reach further than the 7 actin subunits suggested by its stoichiometry. It is also now apparent that troponin itself plays a larger role in steric blocking (Tobacman 2021) and could provide a mechanism for cooperativity along and across the filament. The cryo-EM zebrafish thin filament structure and model we present here correlate highly with the human reconstituted structure. However, our analysis gives rise to further questions about unresolved regions of TnT in zebrafish and human thin filaments, their functions within the macromolecular complex and importantly how mutations that cause cardiomyopathy may affect normal function.

### Electronic supplementary material

Below is the link to the electronic supplementary material.

Supplementary material 1

## Abbreviations

EM: Electron Microscopy
VPP: Volta phase plate
HCM: Hypertrophic cardiomyopathy
TnT: Troponin T
TnC: Troponin C
TnI: Troponin I
