# Supplementary material for: Zebrafish as a model for cardiac disease; Cryo-EM structure of native cardiac thin filaments from Danio Rerio
Source: J Muscle Res Cell Motil. 2023 Jul 22;44(3):179–92. doi: 10.1007/s10974-023-09653-5 (PMC10542308; doi:10.1007/s10974-023-09653-5)
Supplement: Supplementary file 1 — Supplementary material 1 [file 10974_2023_9653_MOESM1_ESM.docx]

**Supplemental Information**

**Table S1. Mincing Solution – Ph 6.8**

| Contents | Concentration |
| --- | --- |
| NaCl | 0.1M |
| MgAc | 5mM |
| EGTA | 2mM |
| Phosphate Buffer | 7mM |
| Protease Inhibitor Cocktail  -AEBSF  -Aprotinin  -Bestatin  -E-64  -Leupeptin  -Pepstatin A | 520 µM  0.4 µM  20 µM  7 µM  10 µM  7.5 µM |

**Table S2. Relaxing Solution – Ph 6.8**

| Contents | Concentration |
| --- | --- |
| NaCl | 0.1M |
| MgAc | 5mM |
| EGTA | 2mM |
| Phosphate Buffer | 7mM |
| Protease Inhibitor Cocktail  -AEBSF  -Aprotinin  -Bestatin  -E-64  -Leupeptin  -Pepstatin A | 520 µM  0.4 µM  20 µM  7 µM  10 µM  7.5 µM |
| ATP | 2.5mM |
| Creatine Phosphate | 10mM |

**Table S3. Microscope Collection and Reconstruction Details**

| **Collection** | **Collection 1 (TF1)** | **Collection 2 (TF2)** |
| --- | --- | --- |
| **No. micrographs** | 3,105 | 2,517 |
| **Magnification** | 81,000x | 81,000x |
| **Pixel size (Å/p)** | 1.048 | 1.048 |
| **Microscope** | Titan Krios | Titan Krios |
| **Detector** | K2 (Counted Mode) | K2 (Counted Mode) |
| **Voltage, kV** | 300 | 300 |
| **Defocus range (µm)** | 0.4 – 1.0 | 0.4 – 1.1 |
| **No. of frames** | 40 | 40 |
| **Dose per frame** | 1.11 | 1.11 |
|  | **Thin Filament Reconstruction (TF1 & TF2)** | |
| **Box Size (p)** | 500 | |
| **Particles** | 9,821 | |
| **Resolution (Å)** | 15.3 | |
|  | **Actin Reconstruction (TF1)** | |
| **Box Size (p)** | 500 | |
| **Particles** | 420,108 | |
| **Resolution (Å)** | 3.85 | |


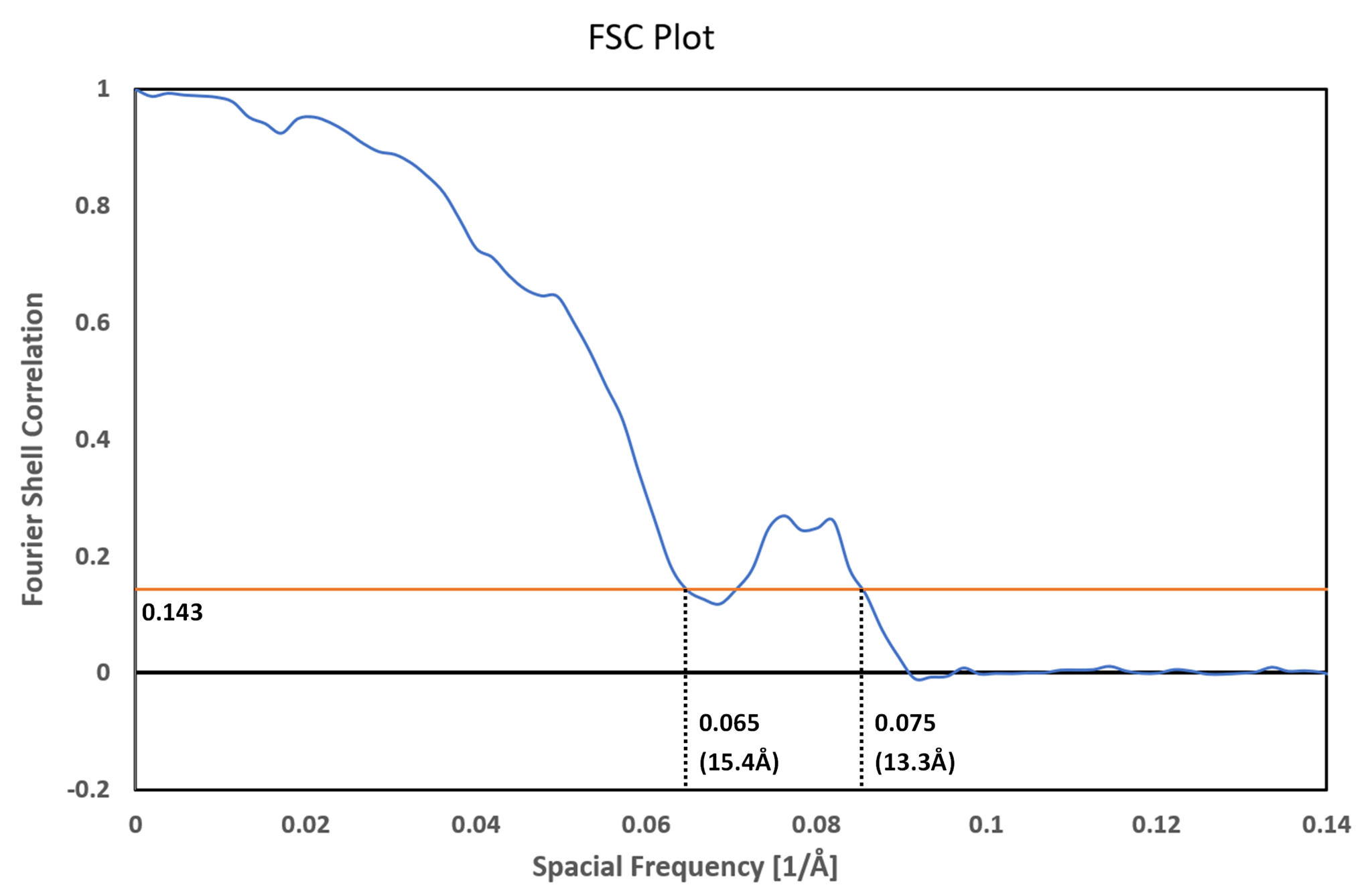


**Fig S1.** Resolution estimation by Fourier shell correlation calculated using independent half maps from zebrafish thin filament structure. A cut-off is shown at 0.143 with the first intersection occurring at spatial frequency 0.065, suggesting a resolution of 15.3Å. The FSC curve only briefly dips below this threshold and later recrosses at 0.075 suggesting a resolution of 13.3Å. This may represent distinct local resolutions within the zebrafish map.

**
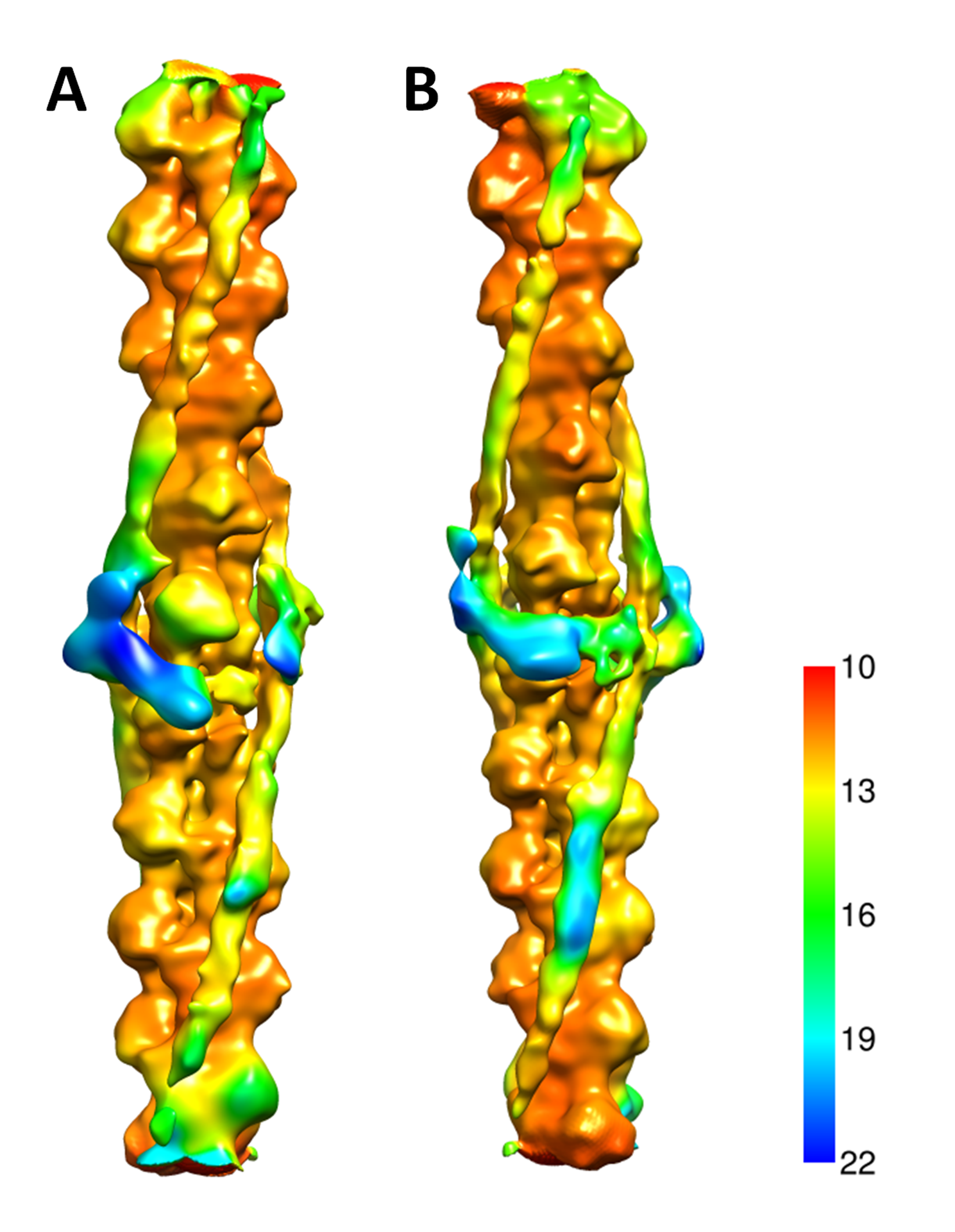
**

**Fig S2** Local resolution 3D map reconstruction obtained using Relion’s Local Resolution function. A & B show the reconstruction from opposing angles to include both troponin complexes. The Map shows local resolution between actin (higher resolution) and accessory proteins (lower resolutions). Performed by inputting half-maps, each generated using subsets of half the particles used in the final Zebrafish reconstruction.


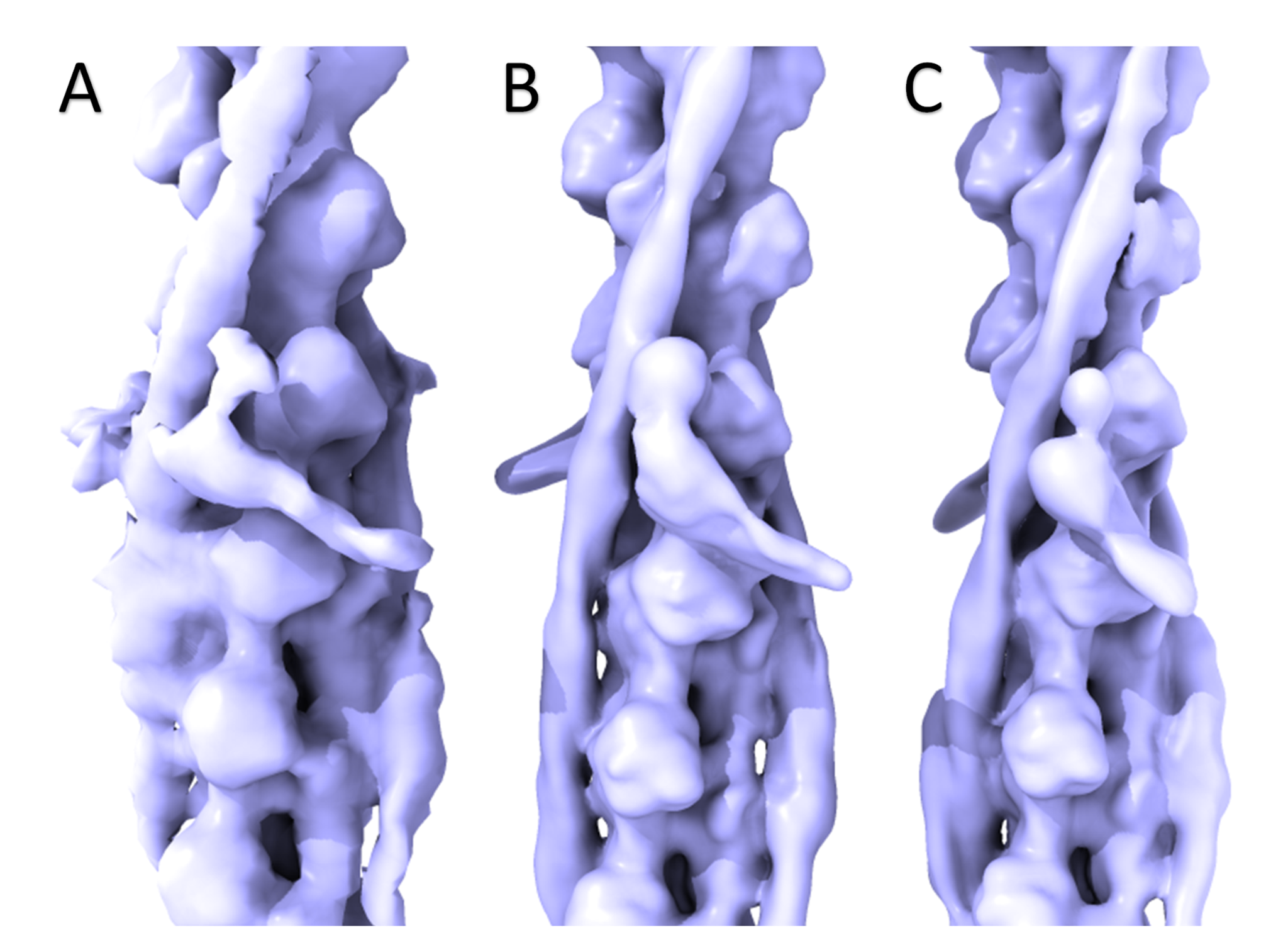


**Fig. S3** Cryo EM reconstructions of the thin filament filtered to a resolution of 15Å. A: The native zebrafish map EMD-15901. B & C: The high and low Ca^2+^human reconstituted thin filament maps EMD-0279 and EMD-0278. The orientation of the troponin core domain in the zebrafish map (A) closely resembles that seen at high Ca^2+^(B).

**
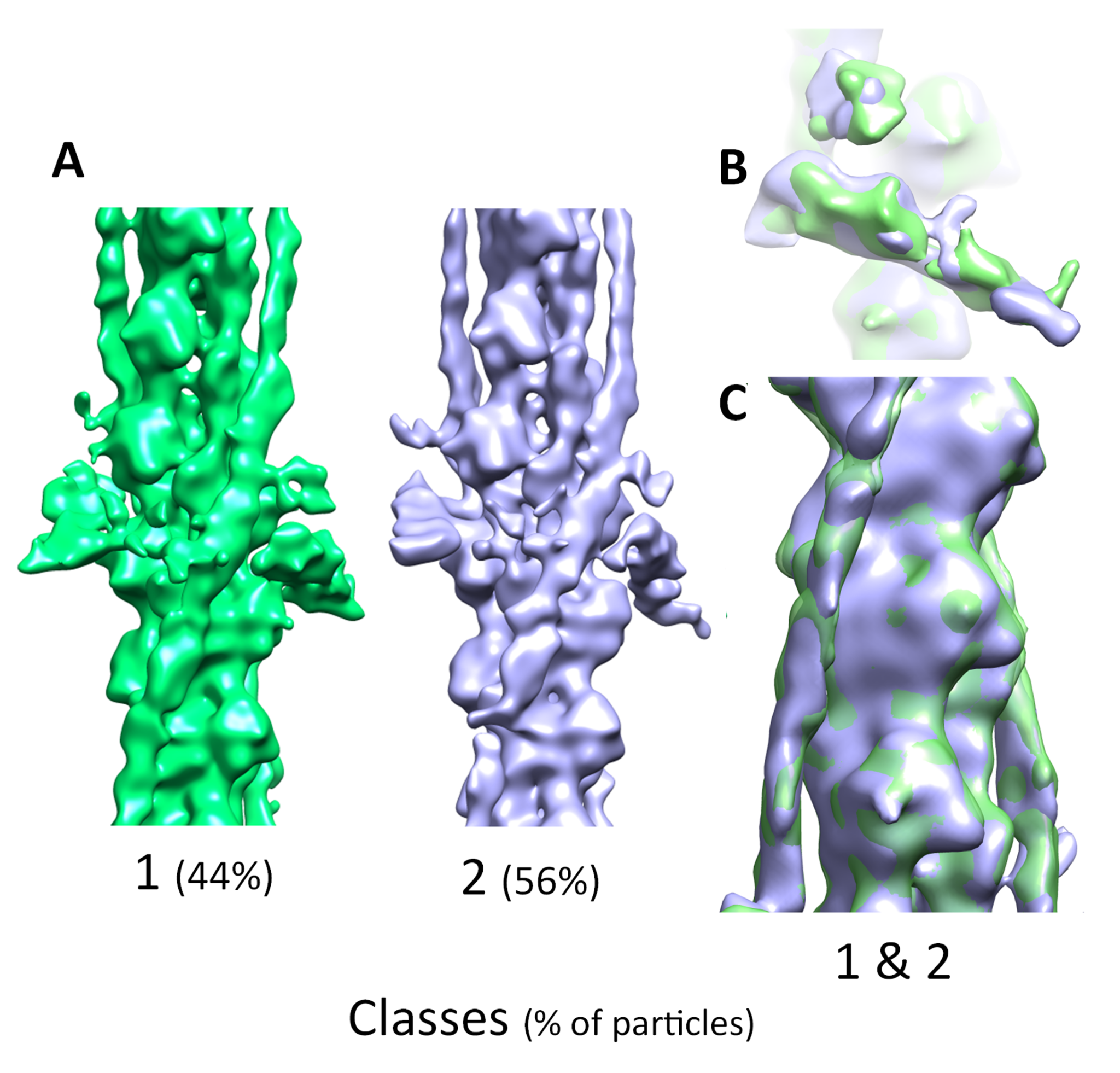
**

**Fig S5** Evaluating the activation state of the data using 3D classification. A: Particle set used for the final zebrafish cryo-EM map used to generate 2 classes in cisTEM. B: Both classes aligned using Chimera’s fit in map function shown superimposed, alignment shows very similar IT arm angle. C: Both classes aligned as in B. with the same tropomyosin position, highlighting a high degree of similarity between the two, suggesting both classes occupy the same activation state.

**
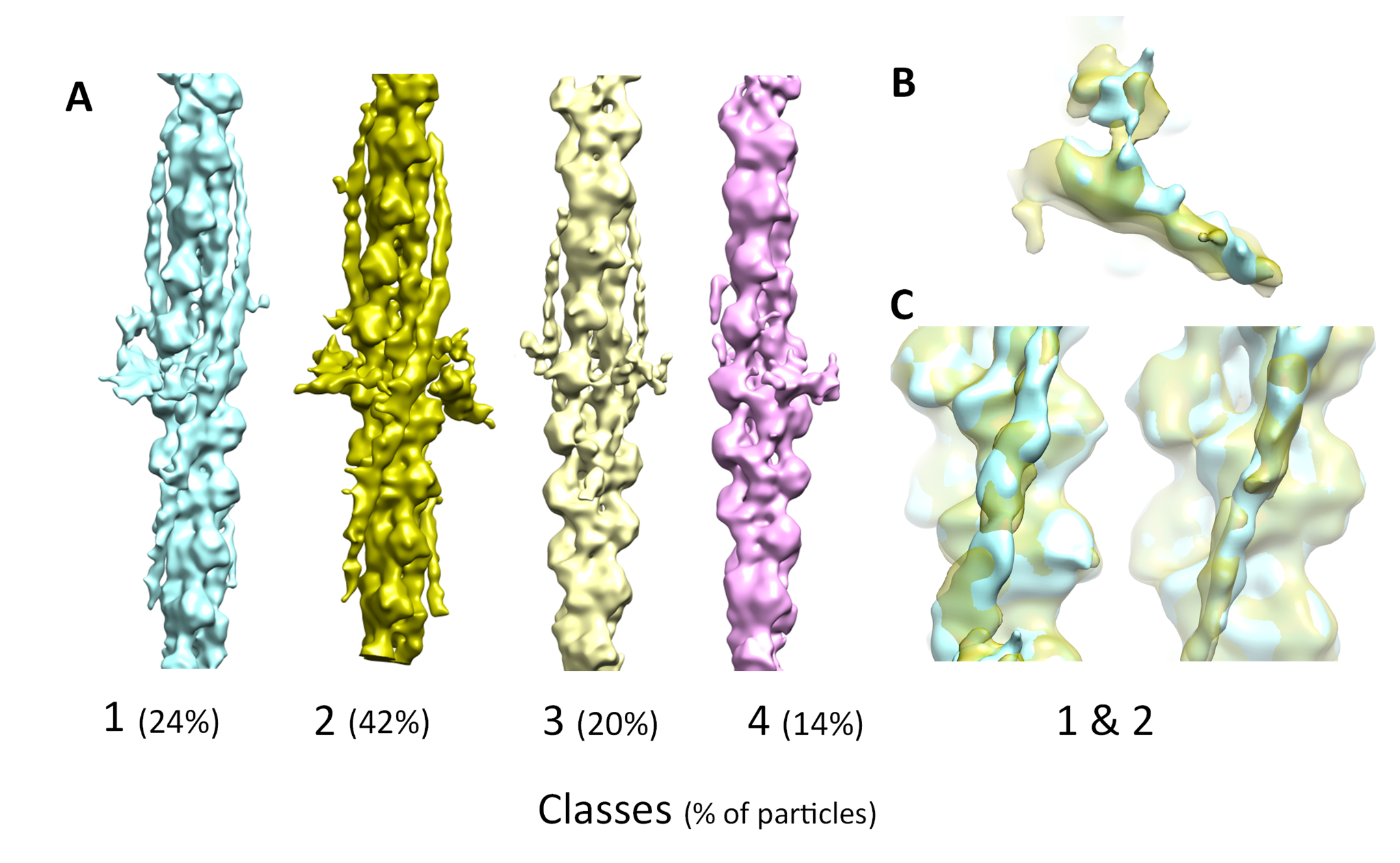
**

**Fig S6** Evaluating the activation state of the data using 3D classification**.** A: Four 3D classes generated in cisTEM from the particles used for the final Zebrafish Cryo-EM map including a subpopulation of thin filaments with single sided troponin decoration (1 & 4) B: Classes 1 & 2 aligned using Chimera’s fit in map function superimposed, alignment shows very similar IT arm angle. C: Classes 1 & 2 aligned as in B. with tropomyosin shown, highlighting a high degree of similarity between the two. Class 3 also showed alignment in the troponin core and tropomyosin with classes 1 & 2, class 4 was lacking sufficient density of either feature to compare. Overall, this suggests classes 1,2 & 3 occupy the same activation state.


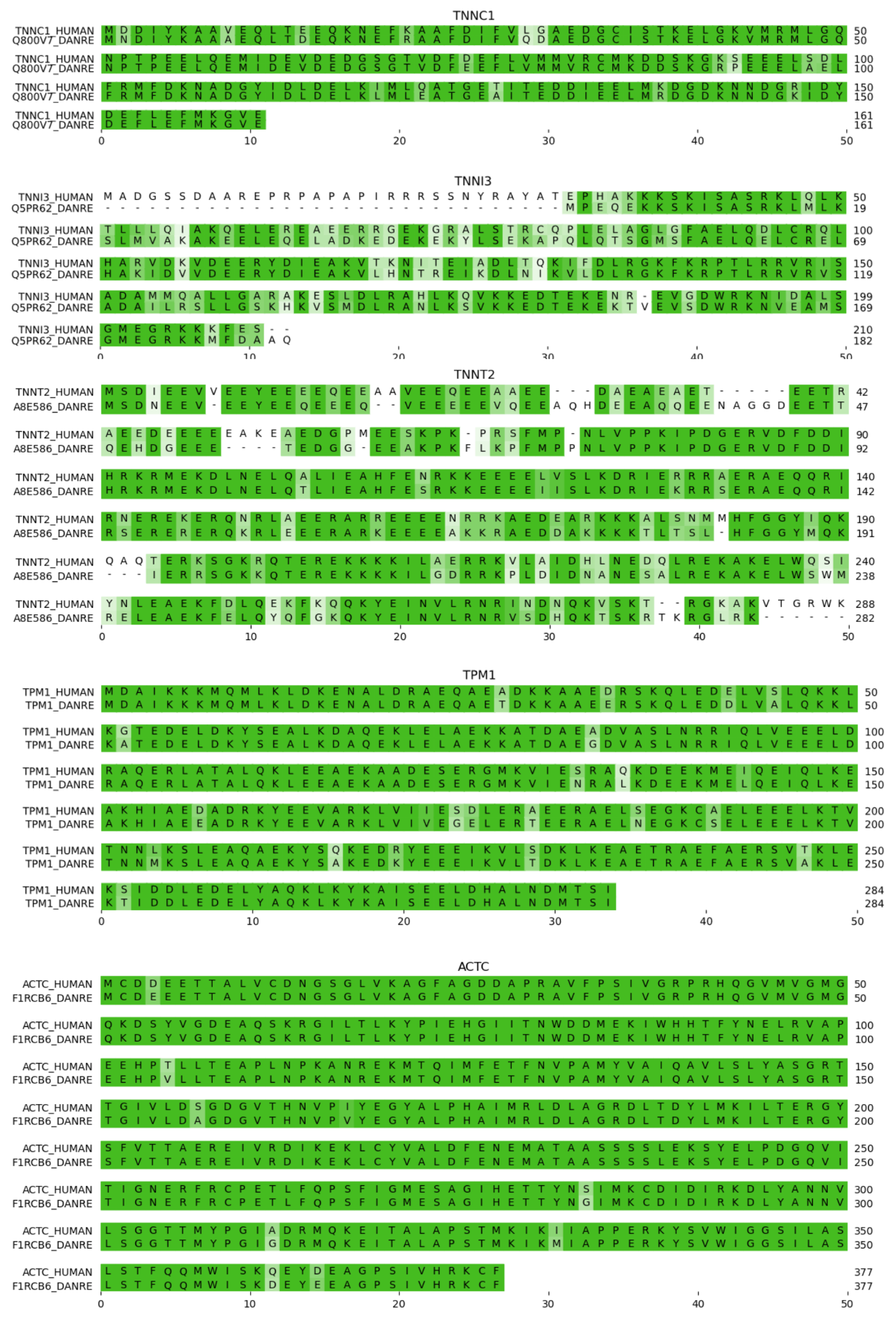


**Fig S7.** A pairwise sequence alignment of the human and zebrafish thin filament genes. Uniprot identifiers TnC: TNNC1_HUMAN & Q800V7_DANRE, TnI: TNNI3_HUMAN & Q5PR62_DANRE, TnT: TNNT2_HUMAN & A8E586_DANRE, Tropomyosin: TPM1_HUMAN & TPM1_DANRE, Actin: ACTC_HUMAN & F1RCB6_DANRE.


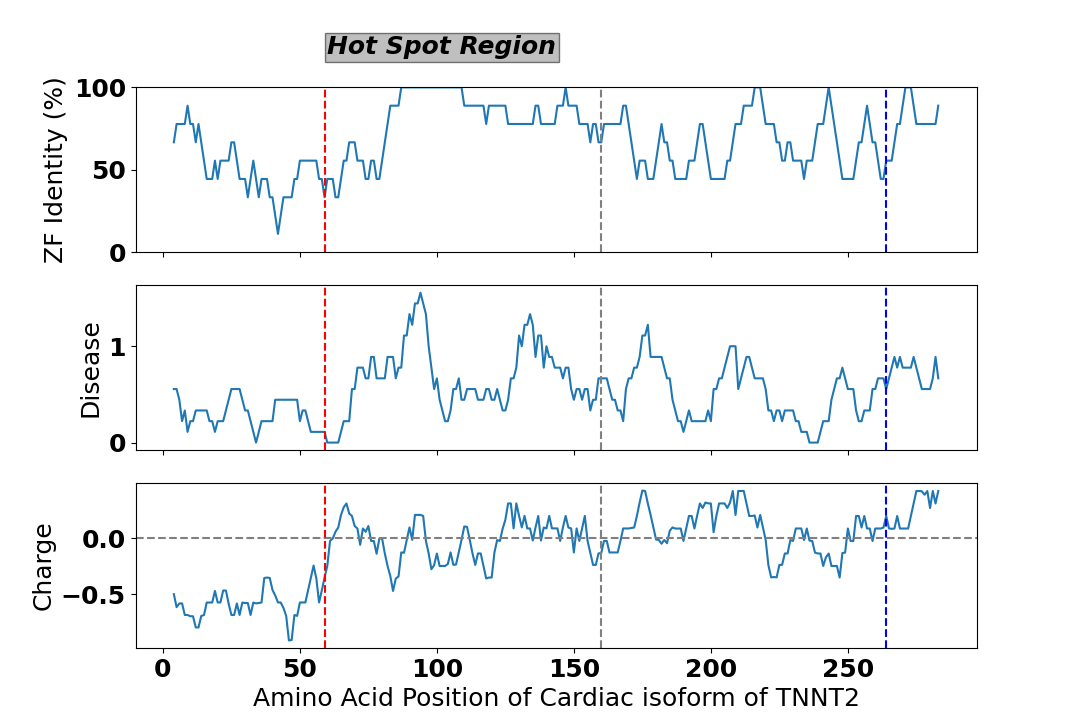


**Fig. S8** Analysis of sequence properties for sub-regions of TNT2_HUMAN. A sliding window of 11 amino acids was moved across the sequence and in that window average properties were calculated for: (Top) Protein sequence identity between human and zebrafish; (Middle) Number of distinct disease-causing mutations per residue (distinct based on position and mutant AA); (Bottom) Net Charge at pH7.


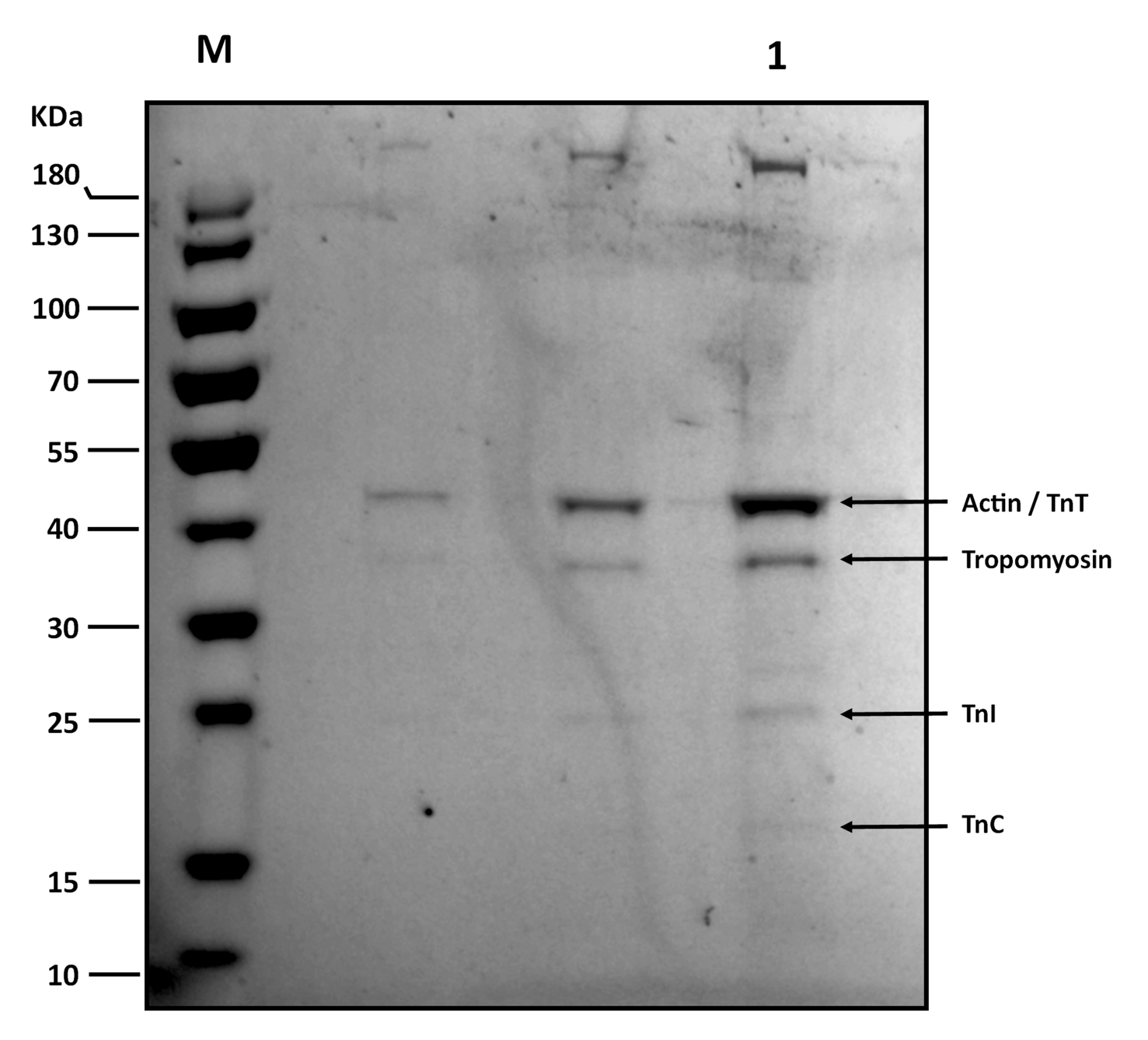


**Fig S9.** SDS-PAGE for zebrafish heart isolation prior to cryo plunge freezing. Lane 1 shows the highest concentration of thin filament isolate providing the clearest bands (compared to middle 2 lanes). Lane M shows the molecular weight markers. Overlapping Actin/TnT band and weaker TnI and TnC bands are consistent with mammalian thin filament gels. (Yamada et al., 2020. Risi et al., 2021)


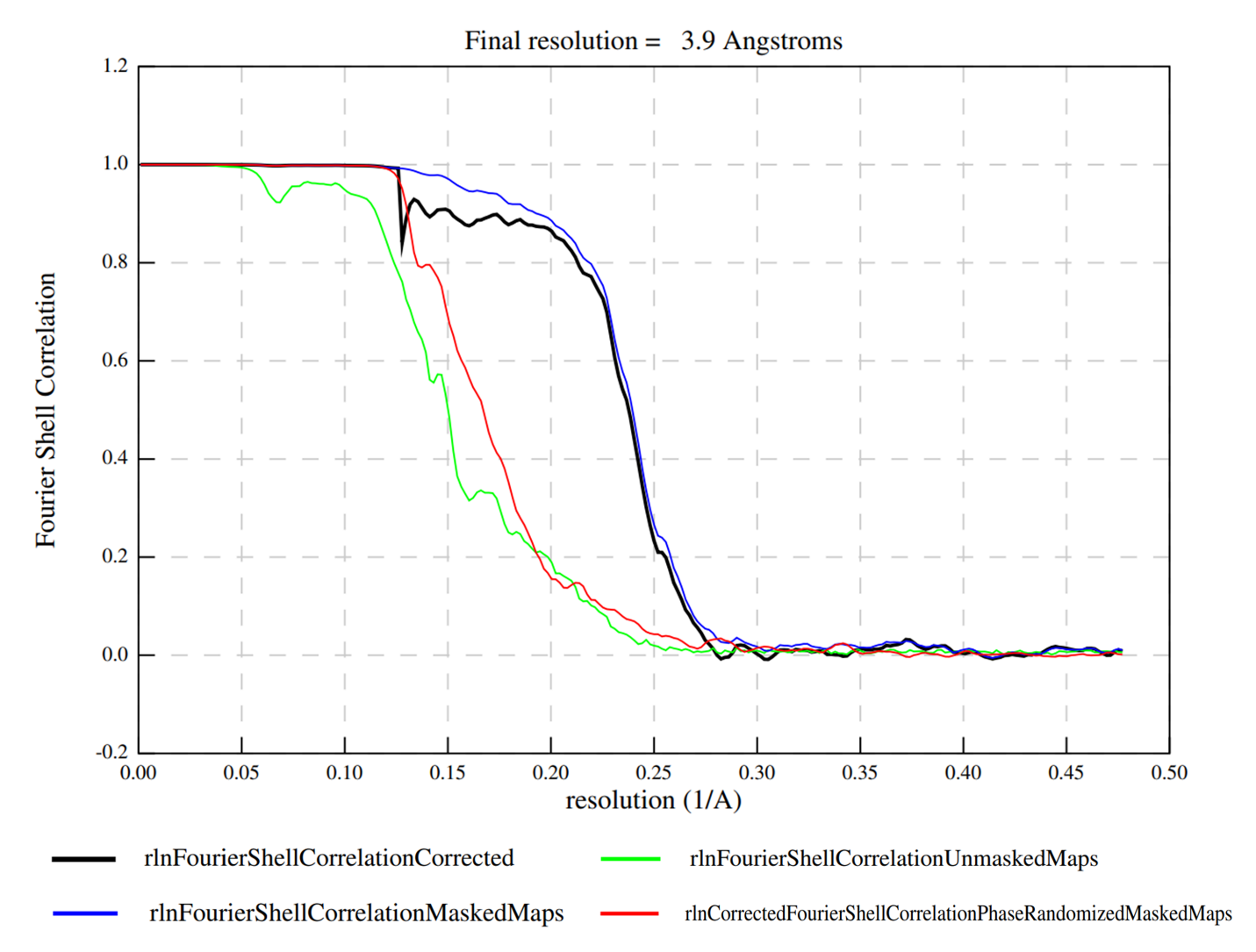


**Fig S10** Resolution estimation by Fourier shell correlation calculated using independent half-maps from zebrafish cardiac actin structure. A cut-off is shown at 0.143 with the first intersection occurring at spatial frequency 0.26 giving a resolution of 3.85Å.


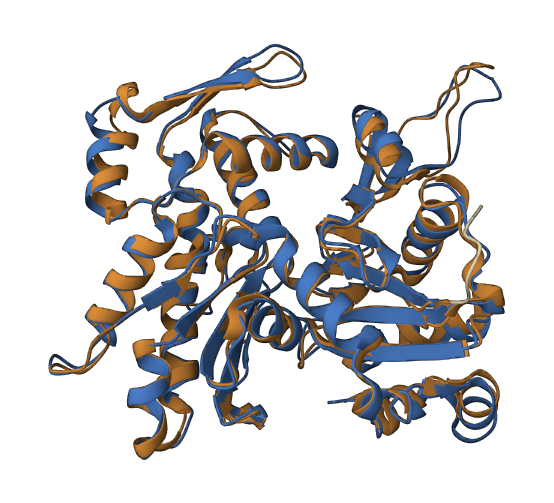


**Fig S11.** Structural similarity between zebrafish actin PDB 8ORD (F1RCB6_DANRE-blue) and human cardiac actin PDB 6KN8 (ACTA_HUMAN-brown) TM score 0.97, RMSD 1.19.
